# The Protective Effects of the Active Fraction of Shaofu Zhuyu Decoction on Hydrogen Peroxide-Induced Oxidative Injury in Vascular Smooth Muscle Cells

**DOI:** 10.3390/molecules15085066

**Published:** 2010-07-26

**Authors:** Li Liu, Jin Ao Duan, Yu Ping Tang, Hong Yue Ma, Shu Lan Su, Jian Ming Guo, Yong Qing Hua

**Affiliations:** Jiangsu Key Laboratory for TCM Formulae Research, Nanjing University of Chinese Medicine, Nanjing 210046, China

**Keywords:** blood stasis syndrome, oxidative damage, reactive oxygen species, apoptosis, vascular smooth muscle cells

## Abstract

In this paper, the protective effects of the active fraction (SF-7) from Shaofu Zhuyu decoction (SFZYD) were tested on a hydrogen peroxide (H_2_O_2_)-induced rat vascular smooth muscle cells (VSMCs) oxidative injury model. This active fraction (SF-7) shows potent antioxidant properties. The cell viability and oxidative damage markers of VSMCs were determined after exposure to H_2_O_2_ for 16 hours. It was observed that SF-7 significantly increased cell survival and reduced apoptosis of H_2_O_2_-injured VSMCs. Moreover, SF-7 could markedly increase intracellular superoxide dismutase (SOD) activity and decrease the malondialdehyde (MDA) level in H_2_O_2_-injured VSMCs, and suppress the generation of intracellular reactive oxygen species (ROS), as well as intracellular Ca^2+^ concentration. Thus, SF-7 exhibits protective effects against H_2_O_2_-injury on VSMCs, which may be associated with its antioxidant properties. It is suggested that SF may be useful in the treatment of blood stasis syndrome in which oxidative injury is mainly implicated.

## 1. Introduction

Shaofu Zhuyu decoction (SFZYD), created by Qingren Wang in the Qing Dynasty of China is a Traditional Chinese Medicine formula for treating blood stasis syndrome. SFZYD consists of ten crude herbs: *Angelica sinensis*, *Ligusticum chuanxiong*, *Paeonia lactiflora*, *Zingiber officinale*, *Cinnamomum cassia*, *Foeniculum vulgare*, *Commiphora myrrha*, *Trogopterus xanthipes*, *Typha angustifolia* and *Corydalis yanhusuo*. This ancient prescription has been used in the clinical treatment of gynecology diseases in China, such as primary dysmenorrheal, menoxenia, pelvic inflammation, etc [1]. A recent study showed that SFZYD could significantly inhibit the constriction of uterine smooth muscle and possess anti-inflammatory activity [2]. A recent study also showed that SFZYD could significantly improve hemorheological indexes of rats in the model of blood stasis and regulation for the function on rat ovary [3].

In our previous study, using the *in vitro* uterine smooth muscle contraction model, an active fraction (SF-7) with obvious inhibitory activity on mouse uterus contraction of SFZYD was isolated [4,5]. It was also found that SF-7 showed other significant activities, such as inhibiting platelet aggregation, promoting rat ovarian granulosa cells proliferation and inhibiting NO of rat peritoneal macrophages. Therefore, the results above implied that SF-7 might be one of the active fractions and contribute to the efficacy of the whole-formula SFZYD.

In blood stasis syndrome, uterine smooth-muscle contraction leads to vascular pressure and the transient ischemic/reperfusion of myometrium and endometrium. As a result, uterine tissue cells generated more reactive oxygen species (ROS). Excessive ROS are able to produce cellular membrane lipid peroxidation, lipid–protein interaction alteration, enzyme inactivation and DNA breakage, and in the end, to cause cell injury (e.g. vascular endothelial cells and vascular smooth cells), apoptosis or necrosis. These detrimental effects are attributed to enhancing intracellular ROS and Ca^2+ ^concentration and to activating inflammatory reactions and apoptotic pathway [6,7]. 

The anti-oxidative and anti-inflammatory actions of the herbs and some compounds in SFZYD had been reported previously [8,9,10,11,12,13,14,15]. In addition, we investigated the protective effects of SF-7 on human umbilical vein endothelial cell (HUVEC) damage induced by adrenaline. The results showed that SF-7 could significantly inhibit the ET release, reverse the NO secretion and promote the PGI_2_ release of HUVEC [16]. In blood stasis syndrome, besides to endothelial cells, vascular smooth muscle cells (VSMCs) are one of the main constituents of the blood vessel wall, and are involved in the maintenance of vessel structure and function. Nowadays, more abundant vascular damages are evidenced by the oxygenation of endothelial cells and smooth muscle cells. Oxygen free-radical attack seems to greatly contribute dominant pathogenesis of vascular disease [17].

Therefore, in the present study, using the oxidative injury model of rat VSMCs induced by hydrogen peroxide (H_2_O_2_), which was widely used as exogenous ROS to produce oxidative stress *in vitro* study [18,19,20,21,22,23]. This paper evaluated the protective effects of SF-7. The aim of this study may provide scientific information to further understanding of the mechanism of action of this formula in blood stasis syndrome.

## 2. Results and Discussion

### 2.1. Effect of SF-7 on viability of H_2_O_2_-injured VSMCs

**Figure 1:**
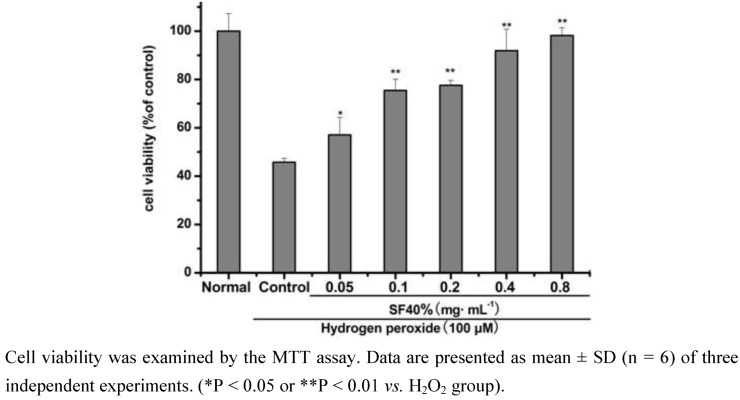
Effect of SF-7 on viability of H_2_O_2_-injured VSMCs.

To determine the effect of SF-7 on H_2_O_2_-induced injury, VSMCs were treated with different concentrations of SF-7 and 100 μM H_2_O_2_ for 16 h. Cell viability was then examined using an MTT mitochondrial function assay. As shown in Figure 1, 100 μM H_2_O_2_ significantly decreased cell viability, which was concentration-dependent attenuated by SF-7 treatment.

### 2.2. Effects of SF-7 on LDH leakage, MDA level and SOD activity in H_2_O_2_-injured VSMCs

A significant increase in LDH release reflecting injury was also observed. The LDH activity was significantly induced by 100 μM H_2_O_2_. As shown in Table 1, the activity of LDH increased to 148.97 ± 18.88% of the normal value. Different concentrations of SF-7 could inhibit the LDH leakage.

**Table 1:** Effects of SF-7 on LDH leakage, intracellular MDA level and SOD activity in H_2_O_2_-injured VSMCs.

Group	LDH	MDA	SOD
	(% of normal)		
Normal	100.00 ± 1.70	100.21 ± 4.65	99.36 ± 4.24
Control	148.97 ± 18.88	146.77 ± 4.23	46.28 ± 4.35
0.1 mg·mL^-1^	145.62 ± 4.88	127.83 ± 16.40*	65.57 ± 2.22**
0.2 mg·mL^-1^	124.00 ± 2.85**	123.09 ± 12.53**	76.48 ± 4.43**
0.4 mg·mL^-1^	107.08 ± 6.29**	119.94 ± 7.23**	96.31 ± 3.81**

The culture medium from each treatment was collected and the LDH activity was analyzed. The intracellular MDA level and SOD activity were determined with spectrophotometry. Data were presented as mean ± SD (n = 6) of three independent experiments. (*P < 0.05 or **P < 0.01 *vs.* H_2_O_2_ group.)

After exposure to 100 μM H_2_O_2_ for 16 h, the intracellular MDA level increased to 146.7 ± 4.23% and SOD activity reduced to 46.28 ± 4.35% of the normal value, suggesting that H_2_O_2_ induced production of thiobarbituric acid reactive substances and inhibited SOD activity. Treatment of the cells with different concentrations of SF-7 lowered MDA level and reversed the decreased SOD activity induced by H_2_O_2_.

### 2.3. Effect of SF-7 on apoptosis in H_2_O_2_-injured VSMCs

**Figure 2:**
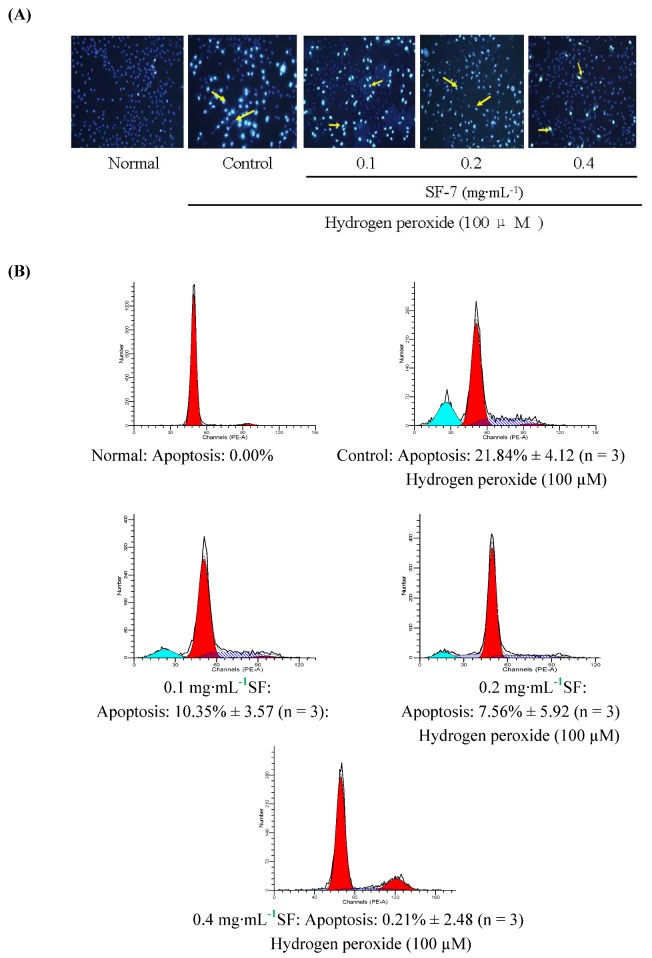
Effect of SF-7 on apoptosis in H_2_O_2_-injured VSMCs. (A) Morphological apoptosis was determined by staining with Hoechst 33342. Arrowheads indicted apoptosis cells. (B) The apoptosis were determined by flow cytometry. Data were presented of three independent experiments.

To observe the effects of SF-7 on H_2_O_2_-induced apoptosis, the cells were stained with Hoechst33342. As shown in Figure 2A, the normal group appeared homogeneous blue fluorescence. But the cells exposed to 100 μM H_2_O_2_ for 16 h displayed blazing blue fluorescence compared to the normal group.SF-7 treatment obviously attenuated VSMCs apoptosis induced by H_2_O_2_. Similarly, as shown in Figure 2B, using flow cytometry, a significant induction of cell apoptosis by H_2_O_2_ were detected, increasing the percentage of apoptotic cells to 21.84% as compared to normal cells. Cell apoptosis induced by H_2_O_2_ was decreased with different concentrations of SF-7.

### 2.4. Effect of SF-7 on intracellular ROS concentration in H_2_O_2_-injured VSMCs

**Figure 3:**
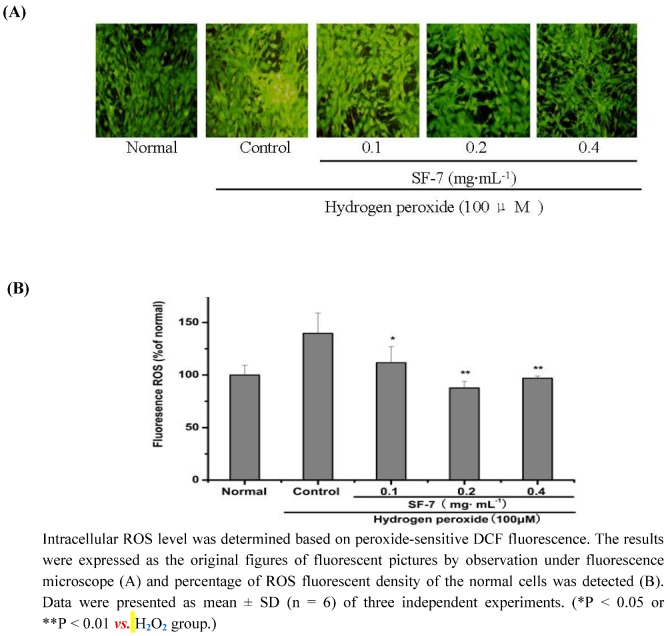
Effect of SF-7 on intracellular ROS in H_2_O_2_-injured VSMCs.

ROS were considered to play an important role in H_2_O_2_-dependent cell death. To elucidate the effect of SF-7 on H_2_O_2_-induced oxidative stress, levels of ROS production in cells were measured using the fluorescence probe DCF. As shown in Figure 3A, and Figure 3B the cells exposed to 100 μM H_2_O_2_ for 16 h displayed increased intensity of DCF-labeled cells. SF-7 treatment could attenuate the increase in fluorescent intensity. SF-7 showed significant inhibition of H_2_O_2_-induced intracellular accumulation of ROS.

### 2.5. Effect of SF-7 on intracellular Ca^2+^ concentration in H^2^O^2^-injured VSMCs

**Figure 4:**
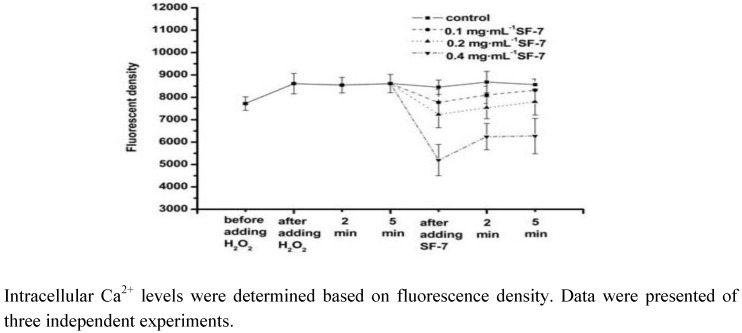
Effect of SF-7 on intracellular Ca^2+^ in H_2_O_2_-injured VSMCs.

As shown in Figure 4, the intracellular Ca^2+^ concentration of the cells increased after exposure to 100 μM H_2_O_2_. Treatment of the cells with SF-7 decreased the intracellular Ca^2+^ concentration especially the high dose group. The result suggested that SF-7 could reduce the intracellular Ca^2+^ concentration in H_2_O_2_-injured VSMCs.

In this study, time-dependent and concentration dependent studies of viability losses in VSMCs induced byH_2_O_2_. SF-7 (0.05–0.8 mg·mL^-1^) showed the most markedly preventive effects on cell injury induced by H_2_O_2_ at 100 μM as compared to other concentrations tested. The magnitude of cell injury peaked at 16 h after H_2_O_2_ exposure. Based on these results, VSMCs were treated with 100 μM of H_2_O_2_ for 16 h.

The present results provide direct and visible evidence of SF-7 for protection against H_2_O_2_-injured VSMCs using the MTT method and LDH leakage assays. In this study, we first demonstrated that the active fraction (SF-7) from an aqueous extract of SFZYD could significantly protect the VSMCs against H_2_O_2_ induced injury. SF-7 significantly prevented the decrease in cell viability and LDH leakage in H_2_O_2_-injured VSMCs. With increasing concentrations of SF-7, the cell viability approached the normal level. 

In the VSMC oxidative injury model, MDA is produced under oxidative stress and reflects oxidative damage of cell membrane and resultant thiobarbituric acid reactive substances, which are proportional to lipid peroxidation and oxidant stress. However, the activity of antioxidant enzymes in cells, such as SOD, for scavenging reactive oxygen species to prevent cell damage, is reduced in oxidative damage. In this study, treatment of VSMCs with H_2_O_2_ caused the decline of SOD activity and increase of MDA level, while incubation of the cells with SF-7 reversed the changes. It could be suggested that the protective effect of SF-7 was related to its antioxidant ability. 

Exposure of VSMCs to H_2_O_2_ also followed an increased VSMCs apoptosis which was attenuated by SF-7, suggesting that SF-7 could act by reduction of apoptosis. Therefore, these results clearly demonstrated that SF-7 exerted protective effects on oxidative damages.

In the vascular system, ischemia injury including inflammation, thrombosis, and angioplasty are accompanied by excessive productions of ROS [24,25,26]. In order to further investigate any relationship between SF-7 inhibition of oxidative damages and the antioxidant properties, ROS generation was assessed in VSMCs treated with SF-7. Addition of SF-7 strongly suppressed the increase in H_2_O_2_ stimulated DCF fluorescence, which indicated strong suppression of intracellular ROS generation. In the present report, after exposure to H_2_O_2_, there was a marked increase of intracellular ROS formation accompanying elevation of intracellular free Ca^2+^ level. It was considered as the result of membrane depolarization leading to the opening of ion channels and increasing Ca^2+^ influx through Ca^2+^ channels. But treatment with SF-7 could significantly reduce the intracellular ROS formation and block H_2_O_2_-induced Ca^2+^ influx. This indicated that SF-7 could attenuate intracellular ROS and Ca^2+^ level [27,28]. Since ROS have been implicated in many pathologic states, it seems possible to speculate that SF-7 might reduce the intracellular Ca^2+ ^level via the antioxidant reaction, or thereby modulating the cellular responses to oxidative injury, but the exact mechanism is not yet clear.

In summary, the results demonstrated that SF-7 inhibited H_2_O_2_ induced injury in VSMCs. These protective effects may be attributed to anti-oxidative actions associated with inhibition of intracellular ROS generation and Ca^2+^ influx. It was reported that SF-7 contained many compounds including paeoniflorin, ferulic acid, typhaneoside, isorhamnetin-3-O-neohesperidin, senkyunolide I and senkyunolide H and quercetin. The preliminary study showed that several compounds may be responsible for its activity [29,30,31]. Therefore, it is necessary to study the antioxidant ability and mechanisms of these compounds in the future. In conclusion, the findings in this study suggested that SF-7 may be used as a feasible alternative therapeutic agent for oxidative damage of the transient ischemic/reperfusion in blood stasis syndrome.

## 3. Experimental

### 3.1. Materials

Dulbecco’s Modified Eagles Medium (DMEM), trypsin, and 3-(4,5-dimethythiazol-2-yl)-2, 5-diphenyltetrazolium bromide (MTT) were purchased from Gibco (Grand Island, NY, USA). Fetal bovine serum (FBS) was from Hangzhou Sijiqing Bioengineering Institute (Hangzhou, Zhejiang, China). Lactate dehydrogenase (LDH), malondialdehyde (MDA), and superoxide dismutase (SOD). Hoechst33342, propidium iodide (PI) and 6-carboxy-2-7-dichlorofluorescein diacetate (DCFH-DA) were provided by Haimen Biyuntian Bioengineering Institute (Haimen, Jiangsu, China). Fluo-4-AM was got Molecular Probe (Eugene, OR, USA). Albiflorin, paeoniflorin, ferulic acid and quercetin were purchased from National Institute for the Control of Pharmaceutical and Biological Products (Beijing, China). Isorhamnetin-3-*O*-neohesperidin, senkyunolide I, senkyunolide H, with 98% purity was provided by Jiangsu Key Laboratory for TCM Formulae Research, Nanjing University of Chinese Medicine, China. SF-7 was freshly prepared as stock solutions in dimethylsulfoxide (DMSO) and diluted with cell culture medium before the experiment. 0.1% (v/v) DMSO had no protective or toxic effect by itself.

### 3.2. Preparation for active fraction SF-7

The mixed crude herbs, *Angelica sinensis, Ligusticum chuanxiong, Paeonia lactiflora, Cinnamomum cassia, Foeniculum vulgare, Zingiber officinale, Commiphora myrrha, Trogopterus xanthipes, Typha angustifolia, and Corydalis yanhusuo* the weight ratio of 3:1:2:1:0.5:1:1:2:3:1(1080, 360, 720, 360, 180, 360, 360, 720, 1080 and 360 g, 5.58 kg total weight) were crushed into small pieces. The mixture was refluxed with water (55.8 L) for 2 h. The filtrates were collected and the residues were then refluxed twice in water (55.8 L) for 1.5 h. Two batches of filtrates were combined.

**Figure 5:**
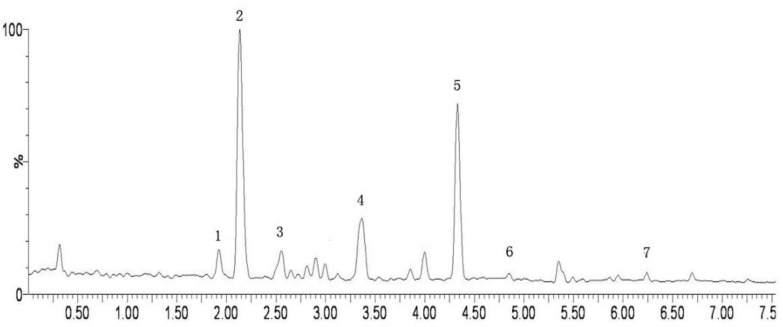
The active fraction of SF-7 in effect UPLC-MS/MS total ion flow diagram (positive ion detection mode). Compounds: 1 = albiflorin, 2 = paeoniflorin, 3 = ferulic acid, 4 = isorhamnetin-3-*O*-neohesperidin, 5 = senkyunolide I, 6 = senkyunolide H, 7 = quercetin).

The solvent was removed below 70 °C to a certain volume at the ratio of 1:1 (w/w, weight of all constituting herbs and the extract filtrates) under vacuum; 95% ethanol was added to the extract filtrates until the concentration of ethanol had been adjusted to 80%. The ethanol solvent was removed below 70 °C to a certain volume of filtrates. The final filtrates were separated by gradient elution with different concentrations of ethanol from macroporous adsorptive resins and then the different fractions obtained. The active fraction, the 40% ethanol elution fraction, which contained many compounds such as albiflorin, paeoniflorin, ferulic acid, isorhamnetin-3-*O*-neohesperidin, senkyunolide I, senkyunolide H, quercetin [32,33] as shown in Figure 5.

### 3.3. Cell culture and drug treatment

Rat VSMCs line was obtained from Institute of Cell Biology, Chinese Academy of Sciences (Shanghai, China). Cells were maintained in high glucose DMEM supplemented with 15% FBS, 100 U·mL^-1^ penicillin, and 100 μg·mL^-1^ streptomycin at 37 °C under an atmosphere of 95% air and 5% CO_2_. The cells were passaged every three days. To study the effects of SF-7 on VSMCs, cells were incubated with SF-7 and H_2_O_2 _at the indicated concentrations for 16 h. The cells underwent the same procedures except SF-7 treatment in H_2_O_2_ group and without both H_2_O_2_ and SF-7 treatments in the normal group. After the cells were cultured for 16 h at 37 °C, the following experiments were performed.

### 3.4. Assay of cell viability with the MTT method

VSMCs (10^4^cells/well in 100 μL medium) were treated with the different concentrations (0.05, 0.1, 0.2 0.4, 0.8 mg·mL^-1^) of SF-7 and 100 μM H_2_O_2 _for 16 h at 37 °C. Then, MTT (20 μL, 0.5 g·L^-1^) was added to each culture well for further incubation. After 4 h, the culture medium was removed and the formazan crystal was dissolved by addition of 150 μL DMSO to each well with vigorously shaking the plate to ensure complete solubilization. Finally, formazan absorbance was assessed by a Multi-detection Microplate Reader (Bio-Tek, USA) at 490 nm.

### 3.5. Detection of LDH leakage, intracellular MDA level and SOD activity with spectrophotometry

After cells were exposed to 100 μM H_2_O_2_ in the presence of the different concentrations (0.1, 0.2, 0.4 mg·mL^-1^) of SF-7 for 16 h, the medium was collected, and the amount of LDH released by cells was determined using an assay kit (Nanjing Jiancheng Co., China) according to the manufacturer’s instruction. The absorbance of samples was read at 440 nm.

Cells were exposed to 100 μM H_2_O_2_ in the presence of the different concentrations (0.1, 0.2, 0.4 mg·mL^-1^) of SF-7 for 16 h. Then the plate was by three freeze/thaw cycles with sonication (10 s, 25 °C) between the cycles [21]. The homogenate was centrifuged at 4,000 rpm at 4 °C for 15 min. Then, SOD activity and MDA level were determined according to the direction of the assay kit (Nanjing Jiancheng Co., China). Thiobarbituric acid reactive substances were assessed by measuring the MDA concentration at 532 nm with the thiobarbituric acid method, which was based on the reaction of MDA with thiobarbituric acid to form a stable chromophoric production. SOD activity was assayed at 550 nm on the basis of its ability to inhibit the oxidation of hydroxylamine by superoxide anion from xanthine-xanthine oxidase system [34,35].

### 3.6. Observation of VSMCs apoptosis by nuclear staining with Hoechst33342

VSMCs (2 × 10^5 ^cells/well in 1000 μL medium) were seeded on poly-L-lysine coated glass cover slips (25 × 25 mm) in six well plates. The cells were exposed to 100 μM H_2_O_2_ and different concentrations (0.1, 0.2, 0.4 mg·mL^-1^) of SF-7 for 16 h and 10 μg·mL^-1^ Hoechst33342 for further 15 min at room temperature. Then the cells were rinsed twice with phosphate-buffered saline (PBS) and imaged with a Fluorescent Microscope (Olympus, JAPAN) in a dark environment within 5 min after the specimen was mounted on the object stage.

### 3.7. Detection of apoptotic cells

Apoptosis was assayed by PI staining followed by analysis with fluorescence-activated cell sorting. The cultured VSMCs cells (10^6^cells/mL) were harvested, washed and fixed with ice-cold alcohol (75%) for more than 24h. After two additional washing, cells were incubated with PBS (pH 7.4) containing RNase (5 U) and PI (50 μg·mL^-1^) for 15 min at 37 °C. Flow cytometry was performed using a FACS vantage SE Flow Cytometer (FACS, Becton Dickinson, USA).

### 3.8. Assay for intracellular ROS

Accumulation of intracellular ROS can be detected using DCFH-DA which crosses cell membranes and is hydrolyzed enzymatically ally by intracellular esterases to non-fluorescent DCFH [35]. In the presence of ROS, DCFH is oxidized to highly fluorescent dichlorofluorescein (DCF), which is readily detected by a fluorescent microplate reader. VSMCs (2 × 10^4 ^cells/well in 100 μL medium) were grown on a black plate for 24 h, and then the cells were exposed to H_2_O_2_ in the presence of the different concentrations (0.1, 0.2, 0.4 mg·mL^-1^) of SF-7. After 16 h, the cells were incubated with 10 μM DCFH-DA in the loading medium in 95% air and 5% CO_2_ for 30 min. DCFH-DA was removed and the cells were rinsed with PBS. The fluorescence was observed by a Fluorescent Microscope (Olympus, Japan). And the fluorescence from each well was captured using a Fluorescent Microplate Spectrophotometer, Spectrum MAX190 (AD, USA) with an excitation wavelength of 488 nm and emission wavelength of 525 nm at 37 °C.

### 3.9. Determination of intracellular Ca^2+^ concentration

The intracellular Ca^2+ ^concentration was determined using fluo-4-AM. The Ca^2+^-sensitive dye fluo-4-AM and pluronic F-127 were separately dissolved in DMSO, and then mixed before the use. VSMCs (2 × 10^4^ cells/well in 100 μL medium) were grown on a black plate. After 24 h, the cells were incubated with 2.5 μM fluo-4-AM in the loading medium in 95% air and 5% CO_2_ for 30 min. Fluo-4-AM was removed and the cells were rinsed with Hank’s solution. The fluorescence from each well was captured using a Fluorescent Microplate Spectrophotometer, Spectrum MAX190 (AD, USA) with an excitation wavelength of 494 nm and emission wavelength of 516 nm at 37 °C. The change of intracellular Ca^2+^ concentration was determined by time course and observed after adding 100 μM H_2_O_2 _and the different concentrations (0.1, 0.2, 0.4 mg·mL^-1^) of SF-7.

### 3.10. Statistical analysis

Statistical Analysis SPSS 12.0 software and Origin 7.0 software were applied to analyze experimental data and results were expressed as means ± S.D. All data were evaluated with analysis of variance (ANOVA) following by Student’s t-test for multiple comparisons and P < 0.05 indicates that the difference was statistically significant.

## 4. Conclusions

SF-7 exhibits protective effect against H_2_O_2_-injury on VSMCs, which may be associated with its antioxidant properties. It is suggested that SF may be useful in the treatment of blood stasis syndrome in which oxidative injury are mainly implicated.

## References

[1] Zhang J., Sun C.L. (2002). Application in gynecology diseases of Shaofu Zhuyu decoction. Chin. J. Nat. Med..

[2] Ye X.L., Wang H., Le J., Chen X. (2002). Effects of Shaofu Zhuyu decoction on uterine convulsion and anti-inflammatory action in rodent. Chin. J. Hosp. Pharm..

[3] Su S.L., Duan J.A. Wang, Hua Y.Q., Tang Y.P. (2008). Evaluating the Effects of Shaofu Zhuyu Decoction on Hemorheology and Ovarian Function in Rat Model of Han-Ning Blood Stasis. Chin. J. Exp. Trad. Med. Form.

[4] Hua Y.Q., Duan J.A., Zhu Q., Wang Q.J. (2007). Study on method of oxytocin induced *in vitro* dysmenorrhea model in mouse. Chin. Pharmacol. Bull..

[5] Su S.L., Hua Y.Q., Duan J.A., Tang Y.P., Lu Y., Ding A.W. (2007). *In vitro* inhibition on the contraction of isolated mouse uterine and chemical components of Shaofu Zhuyu Decoction. J. China Pharm. Univ..

[6] Hool L.C., Corry B. (2007). Redox control of calcium channels: from mechanisms to therapeutic opportunities. Antioxid. Redox Sign.

[7] Heistad D.D. (2006). Oxidative stress and vascular disease. Arterioscler. Thromb. Vasc. Biol..

[8] Zhang Z., Wei T., Hou J., Li G., Yu S., Xin W. (2003). Iron-induced oxidative damage and apoptosis in cerebellar granule cells: attenuation by tetramethylpyrazine and ferulic acid. Eur. J. Pharmacol..

[9] Wu S.J., Ng L.T., Lin C.C. (2004). Antioxidant activities of some common ingredients of traditional Chinese medicine, *Angelica sinensis*, *Lycium barbarum* and *Poria cocos*. Phytother. Res..

[10] Hou Y.Z., Zhao G.R., Yang J., Yuan Y.J., Zhu G.G., Hiltunen R. (2004). Protective effect of *Ligusticum chuanxiong* and *Angelica sinensis* on endothelial cell damage induced by hydrogen peroxide. Life Sci..

[11] Mak D.H., Chiu P.Y., Dong T.T., Tsim K.W., Ko K.M. (2006). Dang-Gui Bu Xue Tang produces a more potent cardioprotective effect than its component herb extracts and enhances glutathione status in rat heart mitochondria and erythrocytes. Phytother. Res..

[12] Kuang X., Yao Y., Du J.R., Liu Y.X., Wang C.Y., Qian Z.M. (2006). Neuroprotective role of Z-ligustilide against forebrain ischemic injury in ICR mice. Brain Res..

[13] Yang X., Zhao Y., Lv Y., Yang Y., Ruan Y. (2007). Protective effect of polysaccharide fractions from Radix *A. sinensis* against tert-butylhydroperoxide induced oxidative injury in murine peritoneal macrophages. J. Biochem. Mol. Biol..

[14] Yang X., Zhao Y., Zhou Y., Lv Y., Mao J., Zhao P. (2007). Component and antioxidant properties of polysaccharide fractions isolated from *Angelica sinensis* (OLIV.) DIELS. Biol. Pharm. Bull..

[15] Yu Y., Du J.R., Wang C.Y., Qian Z.M. (2008). Protection against hydrogen peroxide-induced injury by Z-ligustilide in PC12 cells. Exp. Brain Res..

[16] Su S.L., Yu L., Hua Y.Q., Duan J.A., Deng H.S., Tang Y.P., Lu Y., Ding A.W. (2008). Screening and analyzing the potential bioactive components from Shaofu Zhuyu decoction, using human umbilical vein endothelial cell extraction and high-performance liquid chromatography coupled with mass spectrometry. Biomed. Chromatogr..

[17] Li Y.J., Guan H.D. (2004). Effects of alpinetin on rat vascular smooth muscle cells. J. Asian Nat. Prod. Res..

[18] Li P.F., Dietz R., Harsdorf R. (1997). Differential effect of hydrogen peroxide and superoxide anion on apoptosis and proliferation of vascular smooth muscle cells. Circulation.

[19] Guo F., Zhu B.Y., Chi X.L., Huang H.L. (2007). Inhibition of rosmarinic acid on the apoptosis of vascular smooth muscle cells induced by hydrogen peroxide. Chin. Pharmacol. Bull..

[20] Wu L., Li X.R., Li Y.H., Wang L.J., Tang Y., Xue M. (2009). Proliferative inhibition of danxiongfang and its active ingredients on rat vascular smooth muscle cell and protective effect on the VSMC damage induced by hydrogen peroxide. J. Ethnopharmacol..

[21] Xiao X.H., Liu J.T., Hu J.W., Zhu X.P., Yang H., Wang C.Y., Zhang Y.H. (2008). Protective effects of protopine on hydrogen peroxide-induced oxidative injury of PC12 cells via Ca^2+^ antagonism and antioxidant mechanisms. Eur. J. Pharmacol..

[22] Satpute R.M., Kashyap R.S., Deopujari J.Y., Purohit H.J., Taori G.M., Daginawala H.F. (2009). Protection of PC12 cells from chemical ischemia induced oxidative stress by *Fagonia Arabica*. Food Chem. Toxicol..

[23] Kwok H.H., Ng W.Y., Yang M.S.M., Mak N.K., Wong R.N.S., Yue P.Y.K. (2010). The ginsenoside protopanaxatriol protects endothelial cells from hydrogen peroxide-induced cell injury and cell death by modulating intracellular redox status. Free Radical Biol. Med..

[24] Hearse D.J., Maxwell L., Saldanha C., Gavin J.B. (1993). The myocardial vasculature during ischemia and reperfusion: a target for injury and protection. J. Mol. Cell. Cardiol..

[25] Berliner J.A., Navab M., Fogelman A.M., Frank J.S., Demer L.L., Edwards P.A., Waston A.D., Lusis A.J. (1995). Atherosclerosis: basic mechanisms, oxidation, inflammation, and genetics. Circulation.

[26] Heo S.K., Yi H.S., Yun H.J., Ko C.H., Choi J.W., Park S.D. (2010). Ethylacetate extract from Draconis Resina inhibits LPS-induced inflammatory responses in vascular smooth muscle cells and macrophages via suppression of ROS production. Food Chem. Toxicol..

[27] Kojima K., Kume H., Ito S., Oguma T., Shiraki A., Kondo M., Ito Y., Shimokata K. (2001). Direct effects of hydrogen peroxide on airway smooth muscle tone: Roles of Ca^2+^ influx and Rho-kinase. Eur. J. Pharmacol..

[28] Zhang L., Yu H.X., Sun Y., Lin X.F., Chen B., Chen T., Cao G.X., Wang Z.W. (2007). Protective effects of salidroside on hydrogen peroxide-induced apoptosis in SH-SY5Y human neuroblastoma cells. Eur. J. Pharmacol..

[29] Kanski J., Aksenova M., Stoyanova A., Butterfield D.A. (2002). Ferulic acid antioxidant protection against hydroxyl and peroxyl radical oxidation in synaptosomal and neuronal cell culture systems *in vitro*: structure-activity studies. J. Nutr. Biochem..

[30] Li C.R., Zhou Z., Zhu D., Sun Y.N., Dai J.M., Wang S.Q. (2007). Protective effect of paeoniflorin on irradiation-induced cell damage involved in modulation of reactive oxygen species and the mitogen-activated protein kinases. Int. J. Biochem. Cell Biol..

[31] Kim S.H., Kumar C.N., Kim H.J., Kim D.H., Cho J., Jin C., Lee Y.S. (2009). Glucose-containing flavones—their synthesis and antioxidant and neuroprotective activities. Bioorg. Med. Chem. Lett..

[32] Bao X.J., Su S.L., Duan J.A., Hua Y.Q., Tang Y.P., Shang E.X. (2008). Simultaneous analyze chemical components of inhibiting mice uterine contraction in active fraction of Shaofu Zhuyu Decoction. Chin. J. Exp. Trad. Med. Form.

[33] Zhang H.J., Shen P., Cheng Y.Y. (2004). Identification and determination of the major constituents in traditional Chinese medicine Si-Wu-Tang by HPLC coupled with DAD and ESI-MS. J. Pharm. Biomed. Anal..

[34] Liang Q.M., Yu X.F., Qu S.C., Xu H.L., Sui D.Y. (2010). Acanthopanax senticosides B ameliorates oxidative damage induced by hydrogen peroxide in cultured neonatal rat cardiomyocytes. Eur. J. Pharmacol..

[35] Liu H.T., Li W.M., Xu G., Li X.Y., Bai X.F., Wei P., Yu C., Du Y.G. (2009). Chitosan oligosaccharides attenuate hydrogen peroxide-induced stress injury in human umbilical vein endothelial cells. Pharmacol. Res..

